# The unfolded protein response induced by Tembusu virus infection

**DOI:** 10.1186/s12917-019-1781-4

**Published:** 2019-01-22

**Authors:** Dongmin Zhao, Jing Yang, Kaikai Han, Qingtao Liu, Huili Wang, Yuzhuo Liu, Xinmei Huang, Lijiao Zhang, Yin Li

**Affiliations:** 1 Institute of Veterinary Medicine, Jiangsu Academy of Agricultural Sciences, Jiangsu Province, 210014 People’s Republic of China; 2 Key Laboratory of Veterinary Biological Engineering and Technology, Ministry of Agriculture, Nanjing, Jiangsu Province, People’s Republic of China; 3 Institute of Animal Sciences, Jiangsu Academy of Agricultural Sciences, Jiangsu Province, People’s Republic of China

**Keywords:** Tembusu virus, Endoplasmic reticulum stress, Unfolded protein response, Activation

## Abstract

**Background:**

Tembusu virus (TMUV), classified in the genus *Flavivirus*, causes reduced egg production and neurological problems in poultry. *Flavivirus* replication depends on the host endoplasmic reticulum (ER) and induces ER stress that leads to activation of the cellular unfolded protein response (UPR), an important signalling pathway that regulates many biological functions involved in viral pathogenesis and innate immunity. However, the mechanism of TMUV-induced UPR activation remains unclear.

**Results:**

In this study, we systematically investigated the three UPR pathways in TMUV-infected BHK-21 cells. Our results showed that expression of glucose-related protein 78 (GRP78) and GRP94 was upregulated during the course of TMUV infection. We then demonstrated that TMUV activated the PERK pathway in the early stage of infection, resulting in upregulation of ATF4, GADD34 and CHOP, with CHOP induction leading to caspase-3 activation. We also found the IRE1 pathway to be activated, leading to splicing of X box binding protein 1 (XBP1) mRNA and enhanced expression of p58^IPK^. Finally, we observed increased expression of ATF6 and activity of ER stress-response elements, suggesting stimulation of the ATF6 pathway. In addition, ATF6 pathway activation correlated with the induction of downstream chaperones calnexin, calreticulin, ERp57 and PDI. UPR activity was also observed by the marked elevation in GRP78 and sXBP1 levels in TMUV-infected DF-1 cells.

**Conclusions:**

This is the first report that TMUV infection-induced ER stress activates three branches of the UPR, and these results lay the foundation for elucidating the pathogenesis of TMUV and understanding the inherent mechanism of TMUV infection as well as the host response.

## Background

Tembusu virus (TMUV) is an avian-origin flavivirus that is the causative agent of Tembusu virus disease, a complex pathology of avians associated with a decline in egg production, a decrease in food intake, paralysis and ovarian haemorrhage [1]. Infection and morbidity rates can reach 100%, with mortality of 5–30%, depending on secondary bacterial infections. The prevalence of TMUV has become a severe problem and has seriously hampered the development of the poultry breeding industry in China [2].

As with other *Flaviviridae* viruses, the positive-sense, single-stranded RNA genome of TMUV serves as messenger RNA that is translated into a polyprotein consisting of three structural (capsid, membrane and envelope protein) and seven nonstructural (NS1, NS2a, NS2b, NS3, NS4a, NS4b and NS5) proteins. The polyprotein is subsequently processed into functional products by viral and host proteases. Flaviviruses depend on the endoplasmic reticulum (ER) for their life cycle and are termed ER-tropic viruses [3, 4].

The ER consists of a membranous, interconnected network with complex dynamic construction that is indispensable for various essential cellular processes [5]. For example, various specialized functions, such as calcium homeostasis and intracellular signal transduction, occur in the ER, and it acts as the major site for the synthesis and folding of transmembrane and secreted proteins [6]. Indeed, nearly one-third of proteins in the secretory pathway are folded and mature in the ER [5]. To maintain quality control and ensure the correct folding of secreted proteins, ER homeostasis is tightly regulated, and the accumulation of misfolded proteins in the ER lumen leads to defects in protein folding and modification, a condition known as ER stress [7].

To restore ER homeostasis during ER stress, cells invoke the unfolded protein response (UPR), which aims to decrease the arrival of newly synthesized proteins and to enhance the functional capacity of the ER. The former is achieved via phosphorylation of eukaryotic translation initiation factor 2 (eIF2) and the latter by upregulating the transcription of chaperones and folding enzymes [8]. The UPR consists of three distinct pathways: the protein kinase RNA-like ER kinase (PERK), the inositol-requiring enzyme 1 (IRE1), and the activating transcription factor 6 (ATF6) pathways. In the PERK pathway, PERK is activated by ER stress and phosphorylates the α subunit of eIF2 (eIF2α) at serine residue 51 [9], thereby preventing protein synthesis by repressing mRNA translation. Paradoxically, phospho-eIF2α promotes expression of activating transcription factor 4 (ATF4) [10], a factor that stimulates transcription of C/EBP-homologous protein (CHOP), which in turn induces expression of growth arrest and DNA damage 34 (GADD34) and will target protein phosphatase 1 (PP1) to dephosphorylate eIF2α [11]. Persistent ER stress leads to attenuation or termination of eIF2ɑ phosphorylation and downstream signalling. The IRE1 and/or ATF6 pathway is activated as a result of the restoration of protein synthesis [8]. Activation of the IRE1 (type I ER-resident transmembrane protein) pathway is driven by *trans*-autophosphorylation, whereby phosphorylated IRE1 cleaves the 26-bp intron from the XBP1 (X-boxing binding protein-1) mRNA. This spliced form of XBP1 increases expression of its downstream target genes, including p58^IPK^ and several UPR chaperones [4, 12]. The continuous presence of spliced XBP1 also results in the transfer of UPR signalling from the PERK to the IRE1 and/or ATF6 pathways [12]. During ER stress, ATF6 passes from the ER to the Golgi where it is cleaved by proteases. This active form of ATF6 translocates to the nucleus and activates expression of ER chaperone genes involved in protein folding via binding to ER stress-response elements (ERSEs) [13–16]. Such increased expression of chaperones, including glucose-regulated protein 78 (GRP78), GRP94, calnexin, calreticulin, ER protein 57 kDa (ERp57) and protein disulfide isomerase (PDI), promotes the correct folding of newly synthesized proteins [17]. In unstressed cells, GRP78 binds to the luminal domains of PERK, IRE1 and ATF6 to maintain these proteins in an inactive state [18, 19]. When unfolded or misfolded proteins accumulate in the ER, GRP78 is released from the three UPR sensors and preferentially binds to unfolded or misfolded proteins, resulting in activation of UPR sensors and initiation of UPR signalling cascades [20].

Previous studies have shown that flavivirus infection can activate one or more of the three branches of the UPR. For example, activation of UPR pathways by Dengue virus (DENV) was found to be dependent on the time of infection: early PERK activation and eIF2α phosphorylation were triggered in the early stage of DENV infection; during mid and late stages, the PERK pathway was switched off and the IRE1 and ATF6 pathways activated, respectively [3]. In the case of West Nile virus (WNV), infection with an attenuated strain prevented PERK pathway activation, whereas a highly neurovirulent strain upregulated all three branches of the UPR [21, 22]. As another example, Japanese encephalitis virus (JEV) infection initiated the UPR to promote chaperone expression in cells naturally sensitive to JEV-induced cell death [23]. Additionally, Zika virus (ZIKV) infection, which causes microcephaly in newborns and neurodevelopment abnormalities in adults, activates the IRE1-XBP1 and ATF6 pathways to respond to ER stress in vitro and in vivo [24]. Usutu virus (USUV) infection also induces the UPR, as revealed by the induction of XBP1 mRNA splicing following USUV infection [25, 26]. Furthermore, Tick-borne encephalitis virus (TBEV) triggers IRE1 and ATF6 pathway activation of the UPR, and an IRE1 inhibitor (3,5-dibromosalicylaldehyde) significantly limits TBEV replication in Vero E6 cells, suggesting that this UPR inhibitor might serve as a new therapeutic strategy against TBEV infection [4].

The ER not only provides a platform for flavivirus replication but also for the membrane components for viral particles [25, 27]. Although viral replication leads to the activation of UPR signalling, some facets of the UPR may be beneficial for virus infection. In fact, viruses have evolved to utilize the UPR to promote viral translation and persistence in infected cells [25, 28]. Studies on the DENV envelope protein showed interaction with the ER chaperones GRP78, calreticulin and calnexin, facilitating proper folding and assembly of dengue proteins, and knocking down the expression of these three chaperones resulted in significantly decreased virus production [29]. In addition, by preventing cell death and inhibiting innate immune responses, the ATF6 pathway was found to be necessary for WNV_KUN_ replication, and ATF6 deficiency led to impairment of WNV_KUN_ production, increased CHOP activation and early apoptosis as a result of WNV_KUN_ replication [30].

Despite elementary investigations of the pathogenicity of TMUV [31], the ER stress and UPR induced by TMUV infection remain unclear. Therefore, to explore UPR activation during TMUV infection, we systematically examined induction of the three branches of the UPR and measured the expression levels of UPR-related genes. This work provides new insight into TMUV-induced activation of the UPR.

## Results

### TMUV infection results in the induction of GRP78 and GRP94 expression

**Fig. 1:**
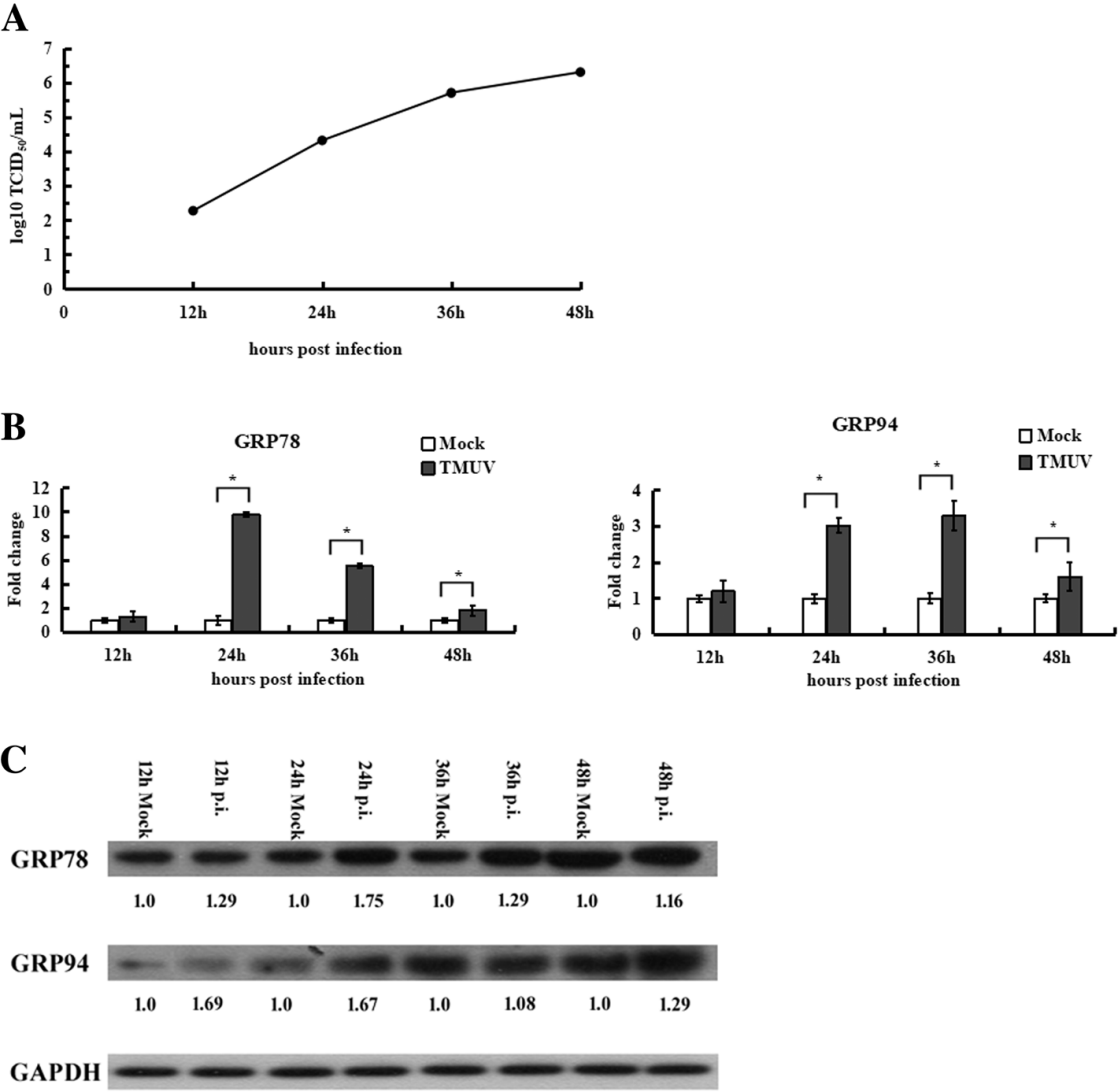
Increased GRP78 and GRP94 expression in TMUV-infected BHK-21 cells. **a**, The replication profile of TMUV in BHK-21 cells. **b**, Expression of GRP78 and GRP94 was determined by real-time RT-PCR. The Y axis represents the fold change of target gene expression in TMUV-infected cells versus that in mock-infected cells. Statistical analyses were performed using Student’s t test in this and in all subsequent figures. The data are expressed as the means±SD of results from three independent experiments. The asterisk indicates a statistically significant difference (*p*<0.05). **c**, Protein levels of GRP78 and GRP94 were examined by western blotting. The intensities of bands were determined using IMAGE J software, and the data represent ratios of TMUV-infected cells to mock-infected cells at the indicated time points

To evaluate the UPR in TMUV infection, the infectivity of TMUV in BHK-21 cells was first determined. Cells were infected with TMUV at an MOI of 3, and viral titres in the supernatant were determined using the plaque assay. The results showed that viral titres increased during the post-infection period, at 48 h (Fig. 1a).

As the UPR is characterized by upregulated expression of chaperones GRP78 and GRP94 [23], the expression levels of GRP78 and GRP94 were analysed during the course of TMUV infection, under the same infection conditions, by real-time RT-PCR and western blotting. As shown in Fig. 1b and c, GRP78 and GRP94 expression was persistently upregulated from 24 to 48 h post-infection compared to mock-infected cells. These results demonstrate that TMUV infection potentially induces ER stress in BHK-21 cells.

### The PERK pathway is activated during TMUV infection

**Fig. 2:**
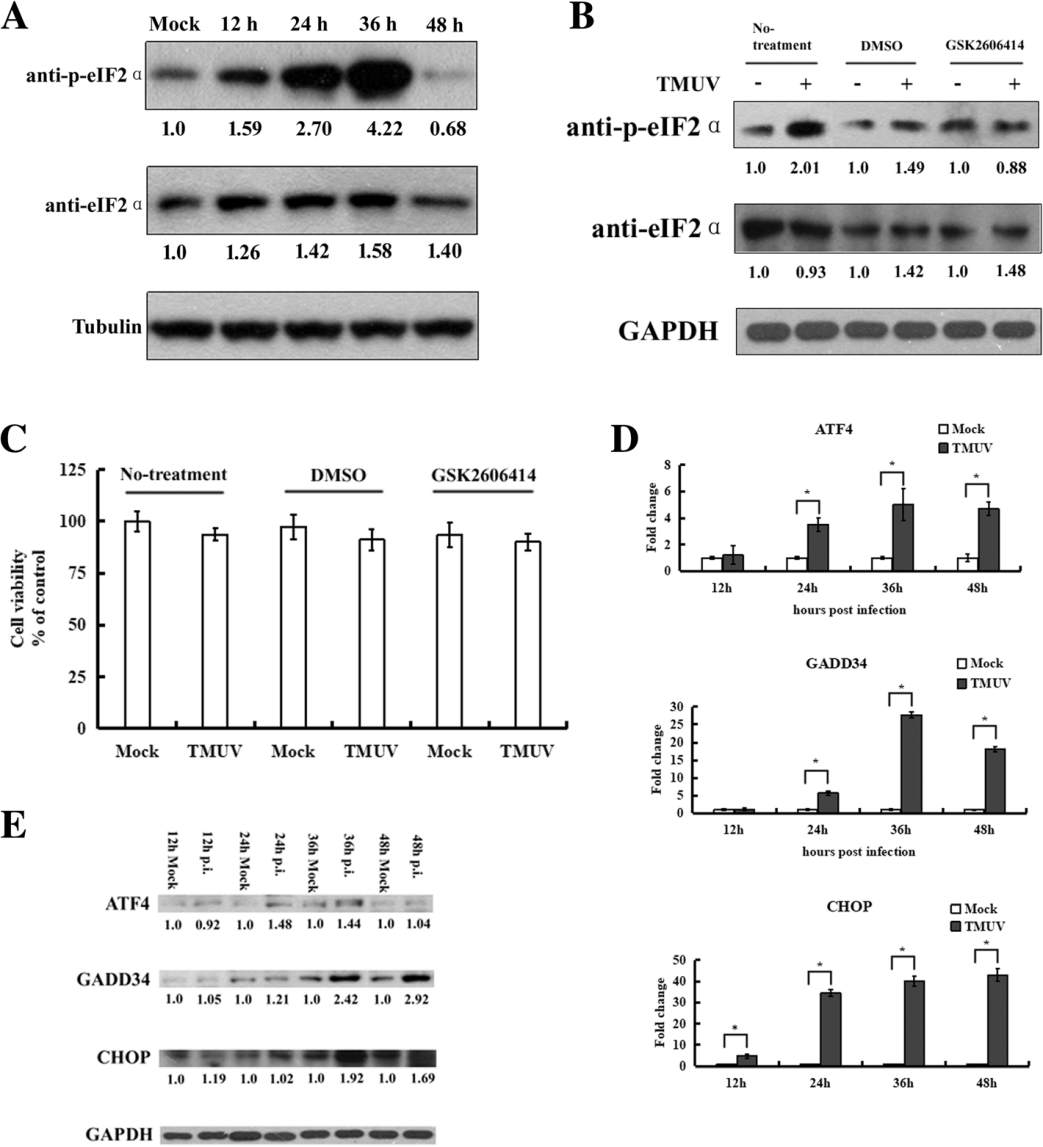
Activation of the PERK pathway by TMUV infection. **a**, Phosphorylation of eIF2α was detected by western blotting. Mock- or TMUV-infected BHK-21 cells were lysed and harvested at the indicated time points. Phosphorylated or total eIF2α was analysed by western blotting using corresponding antibodies. Tubulin was used as an internal control. The intensities of bands were determined using IMAGE J software, and the data represent ratios of TMUV-infected cells to mock-infected cells at the indicated time points. **b**, Inhibition of eIF2α phosphorylation in BHK-21 cells by treatment with the PERK inhibitor GSK2606414. Cells were harvested at 24 h post-infection and subjected to western blotting. The intensities of phospho-eIF2α and eIF2α were determined using IMAGE J software, and the results are shown as ratios of TMUV-infected cells to mock-infected cells. **c**, BHK-21 cells were infected with TMUV at an MOI of 3 and treated with 1 μM GSK2606414 or the corresponding level of DMSO or were not treated. Untreated mock-infected BHK-21 cells were used as a control. Twenty-four hours post-infection, cell viability was assessed using the CCK-8 kit. The data are expressed as the means±SD of results from three independent experiments. **d**, Real-time RT-PCR analysis of components of the PERK pathway during a time course of TMUV infection. The Y axis shows the fold change of target gene expression in TMUV-infected cells, as determined using the comparative CT method. The data are expressed as the means±SD of results from three independent experiments. * *p*<0.05 vs. mock-infected cells. **e**, Protein levels of ATF4, GADD34 and CHOP were examined by western blotting. The intensities of bands were determined using IMAGE J software, and the data represent ratios of TMUV-infected cells to mock-infected cells at the indicated time points

To determine the phosphorylation status of eIF2α during TMUV infection, phosphorylated and total eIF2α were detected by western blotting for 48 h. As shown in Fig. 2a, compared with mock-infected cells, the levels of phosphorylated eIF2α in infected cells increased significantly at 12 h post-infection, reached a peak at 36 h and then decreased to a level lower than that of the mock-infected cells at 48 h post-infection. No significant change in tubulin or total eIF2α expression was observed in either mock- or TMUV-infected cells. Four eIF2α kinases are involved in eIF2α phosphorylation: PERK, double-stranded RNA protein kinase R (PKR), heme-regulated inhibitor kinase (HRI) and general control non-derepressible-2 (GCN2), which are activated to respond to the accumulation of unfolded proteins, the presence of double-stranded RNA during viral infection, heme deficiency and amino acid starvation, respectively [3, 8]. To further clarify whether eIF2α phosphorylation is mediated by PERK, the selective PERK inhibitor GSK2606414 was employed to investigate the role of PERK during the early stage of TMUV infection. The results showed that GSK2606414 reduced the level of phospho-eIF2α at 1 μM, the dose completely inhibiting PERK activity [32] (Fig. 2b). As treatment with GSK2606414 might affect cell viability and ultimately impact experimental results, the CCK-8 assay was performed, and we found that GSK2606414 did not significantly affect the viability of BHK-21 cells (Fig. 2c). The above results suggested that PERK is required for the phosphorylation of eIF2ɑ and that the PERK pathway is stimulated in response to TMUV-induced ER stress.

**Fig. 3:**
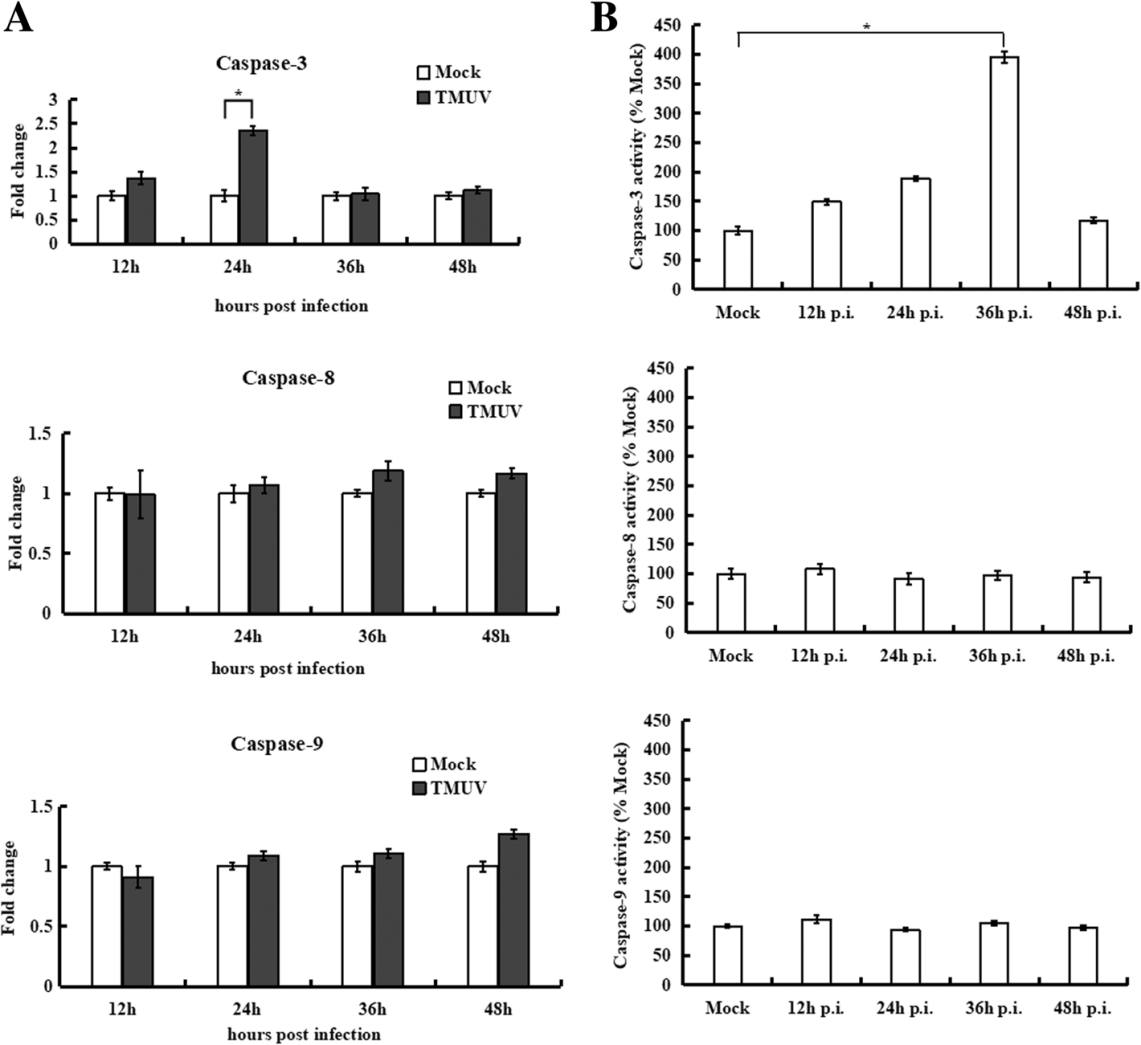
Analysis of caspases in TMUV-infected BHK-21 cells. **a**, Transcriptional expression of caspases was determined by real-time RT-PCR. The Y axis shows the fold change of target gene expression in TMUV-infected cells, as determined by the comparative CT method. **b**, Caspase activity was determined using colorimetric assays. The data are expressed as the means±SD of results from three independent experiments. * *p*<0.05 vs. mock-infected cells

Previous studies have demonstrated that persistent eIF2α phosphorylation selectively increases expression of ATF4 [10]. In this study, ATF4 expression increased significantly after 12 h post-infection, resulting in the induction of CHOP and in turn expression of GADD34 (Fig. 2d and e). CHOP is considered an important specific factor in the ER stress-induced apoptosis pathway [33], and the transcriptional expression and activity of caspase-3, − 8 and − 9 were determined using real-time RT-PCR and enzyme assay kits, respectively. As shown in Fig. 3, no evident change in caspase-8 and caspase-9 occurred in cells at 48 h post-infection compared to mock-infected cells. However, a similar trend for caspase-3, remaining unchanged at 24 h post-infection but increasing significantly at 36 h post-infection, compared to the mock-infected cells was observed. These data suggest that TMUV infection leads to the induction of apoptosis and that ER stress-mediated apoptotic signals ultimately converge on caspase-3.

### TMUV infection activates the IRE1 pathway

**Fig. 4:**
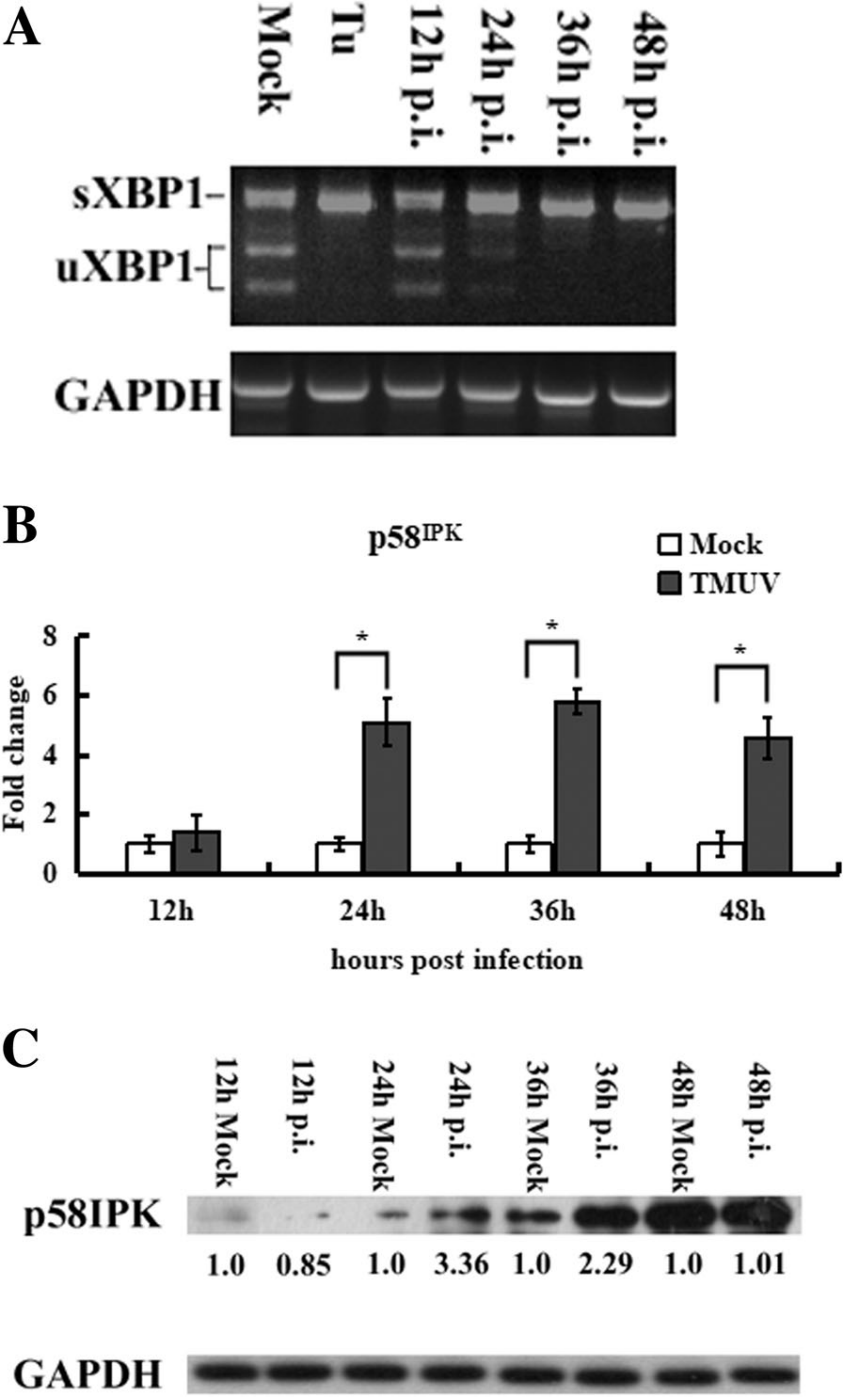
TMUV infection induces XBP1 mRNA splicing and leads to p58IPK upregulation. **a**, XBP1 mRNA was amplified by RT-PCR and digested by Pst I. BHK-21 cells were mock-infected or infected with TMUV. Cells treated with 1 μM tunicamycin (Tu) for 12 h were used as a positive control. At the indicated times after infection, XBP1 mRNA was amplified by RT-PCR using XBP1-specific primers, after which the XBP1 fragment was subjected to Pst I digestion, and the products were separated by 1% agarose gel electrophoresis. **b**, p58IPK upregulation was detected by real-time RT-PCR. The Y axis shows the fold change of target gene expression in TMUV-infected cells, as determined by the comparative CT method. The data are expressed as the means±SD of results from three independent experiments. * *p*<0.05 vs. mock-infected cells. **c**, The protein level of p58IPK was examined by western blotting. The intensities of bands were determined using IMAGE J software, and the data represent ratios of TMUV-infected cells to mock-infected cells at the indicated time points

In the IRE1 pathway, autophosphorylated IRE1 removes a 26-bp intron from the unspliced XBP1 (uXBP1) mRNA, the translation of which produces a stable, active 371-amino acid isoform, spliced XBP1 (sXBP1), that binds to the ERSE sequence of many UPR target genes, leading to transcription of ER-chaperone proteins [34]. Splicing of uXBP1 results in loss of the Pst I restriction site located in the intron [35]. At 36 h and 48 h post-infection, the 530-bp RT-PCR products from TMUV-infected cells were found to be resistant to Pst I digestion, whereas at 12 h and 24 h post-infection, the RT-PCR products from TMUV-infected cells were digested by Pst I to generate 317-bp and 213-bp fragments (Fig. 4a). p58^IPK^ is a downstream gene transcriptionally induced by sXBP1 [36], and as shown in Fig. 4b and c, significantly increased transcription of p58^IPK^ was detected in cells infected with TMUV. The results above show that TMUV activates the IRE1 pathway.

### TMUV infection activates the ATF6 pathway

**Fig. 5:**
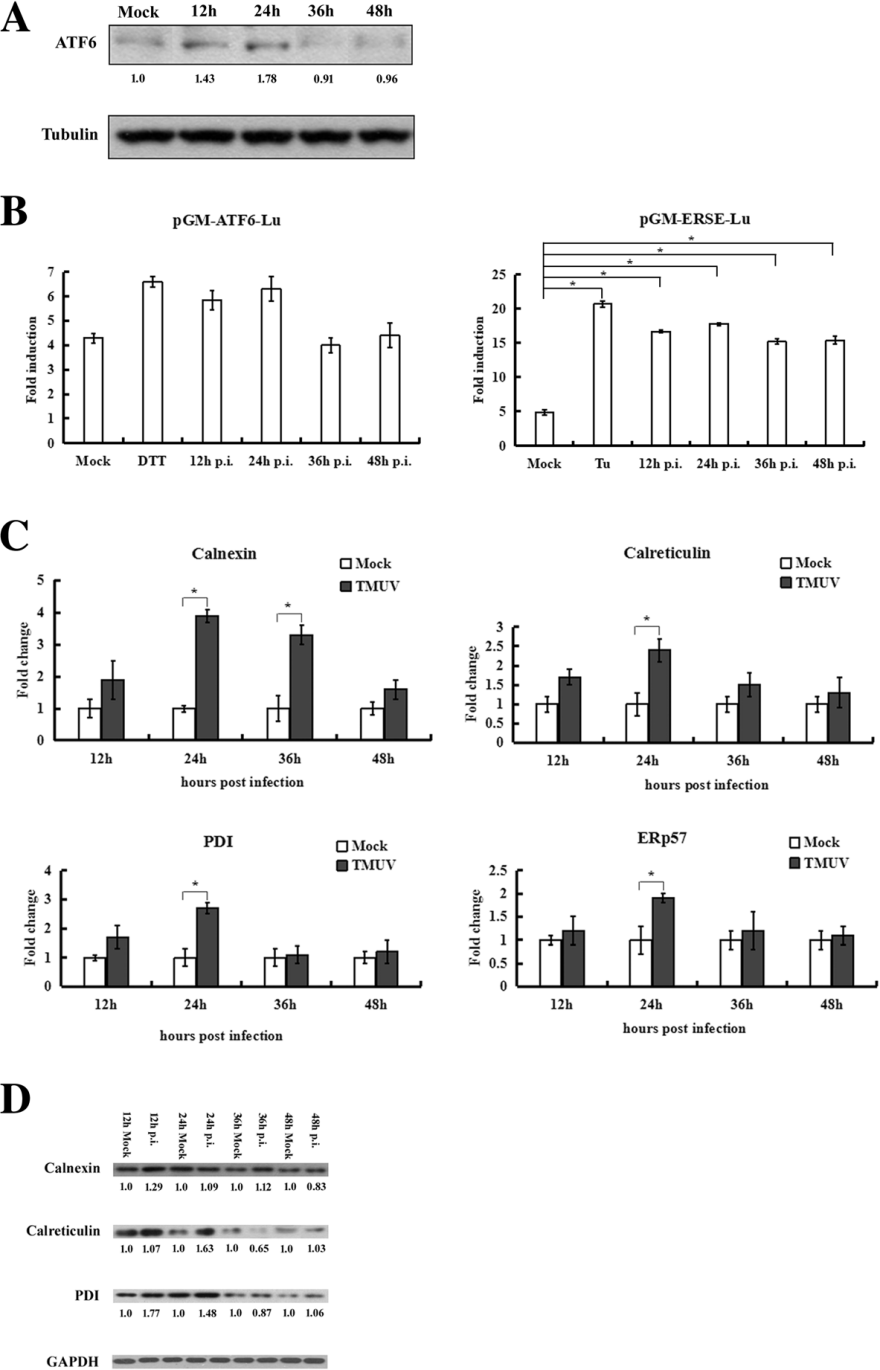
Analysis of the ATF6 pathway during TMUV infection. **a**, Expression of ATF6 after TMUV infection was examined by western blotting using an antibody specific for full-length ATF6. Tubulin was used as an internal control. The intensities of bands were determined using IMAGE J software, and the data represent ratios of TMUV-infected cells to mock-infected cells at the indicated time points. **b**, Activation of the ATF6 pathway was monitored by a dual luciferase reporter gene assay. BHK-21 cells were transfected with pGM-ATF6-Lu or pGM-ERSE-Lu. Firefly luciferase activity was normalized based on Renilla luciferase activity in cells cotransfected with pRL-TK. After 48 h of transfection, the cells were mock infected or infected with TMUV. Cells treated with 1 μM tunicamycin for 12 h or 2.5 mM dithiothreitol (DTT) for 7 h were used as a positive control. Cells were collected at the indicated time points and assayed for firefly and Renilla luciferase activities. The values represent the means±SD of results from three independent experiments. **c**, Expression of chaperones induced by TMUV infection. The Y axis shows the fold change of target gene expression in TMUV-infected cells, as determined by the comparative CT method. The data are expressed as the means±SD of results from three independent experiments. * *p*<0.05 vs. mock-infected cells. **d**, Protein levels of chaperones were examined by western blotting. The intensities of bands were determined using IMAGE J software, and the data represent ratios of TMUV-infected cells to mock-infected cells at the indicated time points

During ATF6 activation, the full-length protein (90 kDa) is cleaved to release an active 50-kDa variant of ATF6 comprising the N-terminus [37, 38]. To further explore whether TMUV infection induces the ATF6 branch of the UPR, ATF6 expression was detected by western blotting using an antibody against full-length ATF6. As shown in Fig. 5a, TMUV infection slightly increased the level of ATF6 at 12 h and 24 h post-infection. A dual-luciferase reporter assay was then carried out to evaluate the transcriptional activity of ATF6 and ERSE. Forty-eight hours after transfection, cells were infected with TMUV, and the results showed slightly increased (1.5-fold) luciferase activity in cells transfected with pGM-ATF6-Lu compared with that of mock-infected cells. In addition, cells transfected with pGM-ERSE-Lu exhibited a 3.8-fold increase in luciferase activity compared with that in mock-infected cells (Fig. 5b). Furthermore, expression of ATF6-induced chaperones, including calnexin, calreticulin, ERp57 and PDI, was analysed by real-time RT-PCR and western blotting. Compared with cells mock-infected with TMUV, expression of ATF6-induced chaperones was upregulated from 12 h to 24 h post-infection and then returned to the level of the mock control (Fig. 5c and d). These results indicate that TMUV infection activates the ATF6 pathway.

### UPR induction in TMUV-infected DF-1 cells

**Fig. 6:**
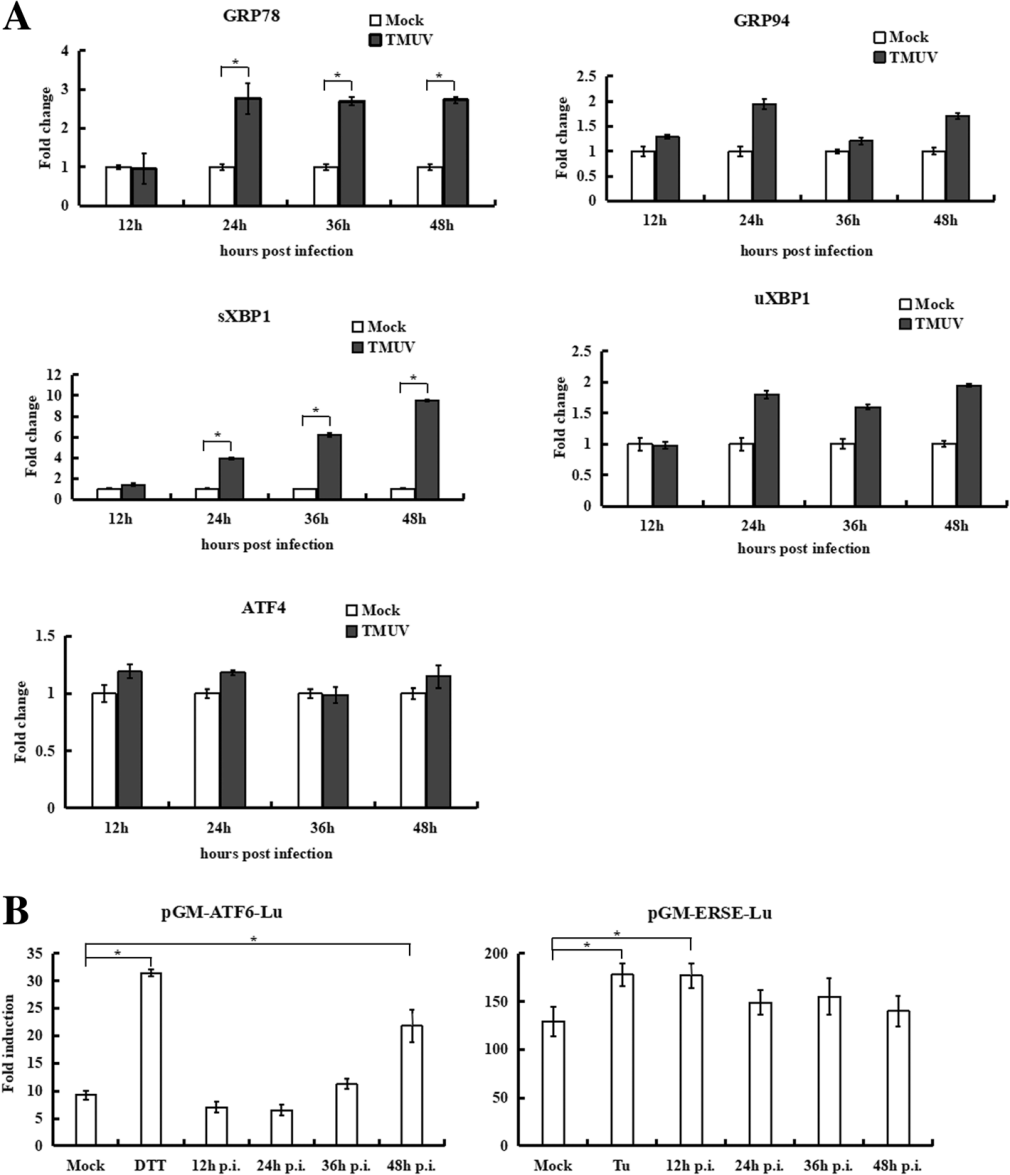
The UPR is induced by TMUV infection in DF-1 cells. **a**, Expression of GRP78, GRP94 sXBP1, uXBP1 and ATF4 was determined by real-time RT-PCR. The Y axis represents the fold change of target gene expression in TMUV-infected cells versus that in mock-infected cells. **b**, Activation of the ATF6 pathway in TMUV-infected DF-1 cells was monitored by a dual luciferase reporter gene assay. DF-1 cells were transfected with pGM-ATF6-Lu or pGM-ERSE-Lu. Firefly luciferase activity was normalized based on Renilla luciferase activity in cells cotransfected with pRL-TK. After 48 h of transfection, the cells were mock infected or infected with TMUV. Cells treated with 1 μM tunicamycin for 12 h or 2.5 mM dithiothreitol (DTT) for 7 h were used as a positive control. Cells were collected at the indicated time points and assayed for firefly and Renilla luciferase activities. The values represent the means±SD of results from three independent experiments. The asterisk indicates a statistically significant differences (*p*<0.05)

Considering that TMUV is an avian pathogen, UPR induction was further assessed in TMUV-infected DF-1 cells by real-time RT-PCR (Fig. 6). At 24 h post-infection, expression of GRP78 and GRP94 showed a significant (3-fold) induction and a near 2-fold increase, respectively. Using primers that specifically detect the spliced or unspliced form of XBP1, we found that XBP1 splicing was increased approximately 10-fold at 48 h post-infection; only a modest induction (~ 2-fold increase) of unspliced XBP1 was observed at 24 h post-infection. Conversely, no ATF4 expression change was observed over a 48-h time course. The results of the dual luciferase reporter assay demonstrated that transfection with pGM-ATF6-Lu resulted in a 2.4-fold increase in luciferase activity at 48 h post-infection compared with the activity of mock-infected cells. At 12 h post-infection, the cells transfected with pGM-ERSE-Lu exhibited a 1.4-fold increase in luciferase activity compared with that in mock-infected cells. These data suggest that TMUV induction of the UPR in DF-1 cells primarily occurs via the IRE1 pathway, with some induction of the ATF6 pathway.

## Discussion

The viral life cycle consists of a series of events involving entry, protein synthesis and modification, genome replication and maturation. Viruses utilize the mechanisms and resources of host cells to complete their entire replication cycle [39]. During this process, quantities of viral proteins are synthesized within infected cells, leading to the accumulation of unfolded or misfolded proteins in the ER and resulting in the activation of ER stress [4]. To cope with ER stress, three sensors, PERK, IRE1 and ATF6, are stimulated to reduce expression of new proteins, to facilitate the folding of proteins and to enhance the degradation of misfolded proteins, also known as the UPR [18].

*Flavivirus* particles are assembled and mature in the ER lumen [40], and several flaviviruses, including JEV, DENV, WNV and TBEV, have been reported to stimulate the UPR to lessen the ER stress caused by the accumulation of viral proteins [4, 21, 41, 42]. In addition, viruses have also evolved different strategies to manipulate or customize the UPR for their own benefit [43]. Many studies have reported that the life cycle of flaviviruses is associated with the UPR. For instance, Ambrose and Mackenzie et al. reported that ATF6-deficient cells infected with WNV_KUN_ exhibit a decline in viral protein and infectious virion production. These cells also show increased eIF2α phosphorylation and CHOP transcription, suggesting that decreases in WNV_KUN_ protein production may be attributed to the inability to manipulate the PERK-mediated response [30]. Furthermore, Yu et al. reported that induction of the IRE1 pathway by DENV infection protects cells against apoptosis, alleviates ER stress and facilitates virion production [44]. He et al. reported that classical swine fever virus (CSFV)-induced ER stress promotes viral replication, with a benefit from activation of IRE1-XBP1-GRP78 signalling [43]. These studies indicate the critical role of the UPR in viral infection. However, the ER stress and UPR signalling involved in TMUV-infected cells have not been fully explored. Here, we comprehensively examine the UPR initiated by TMUV infection and report for the first time that TMUV triggers activation of the PERK, IRE1 and ATF6 pathways in BHK-21 cells.

Due to their roles as molecular chaperones, GRP78 and GRP94 are used extensively as biological markers for the onset of ER stress and the UPR. Because the induction of GRP78 and GRP94 is regulated primarily at the transcriptional level, GRP78 and GRP94 mRNA abundance is commonly determined by real-time RT-PCR [45]. In our study, relative gene expression of GRP78 and GRP94 was significantly higher in TMUV-infected cells at 24 h post-infection compared to mock-infected cells. The observed induction of GRP78 and GRP94 is possibly due to the overwhelming expression of viral proteins exceeding normal ER folding capacity, thereby causing ER stress and activating the UPR. Using the viral overlay protein binding assay (VOPBA) and LC-MS/MS analysis, our previous study identified GRP78 as a receptor for TMUV on BHK-21 cells. Furthermore, antibody inhibition and siRNA-mediated knockdown of GRP78 inhibits TMUV infection in BHK-21 cells [46]. Taken together, these findings highlight the multiple critical roles of GRP78 in the life cycle of TMUV.

PERK and eIF2α signalling is involved in the early stage of ER stress [47], and very early during TMUV infection, we observed continuously high levels of phosphorylated eIF2α, indicating activation of the PERK pathway early in infection. GADD34 mediates the dephosphorylation of eIF2α in a negative feedback loop and subsequently promotes recovery from translational attenuation [11]. In this study, robustly enhanced expression of GADD34 was observed at 36 h post-infection. Consistent with this observation, phosphorylation of eIF2α in TMUV-infected cells was suppressed after 36 h post-infection. Furthermore, at 48 h post-infection, the level of eIF2α phosphorylation in TMUV-infected cells was lower than that in mock-infected cells. This lower level of eIF2α phosphorylation indicates elevated translational initiation and higher overall level of protein synthesis at this time point, which are favourable for viral protein synthesis [48].

The UPR is often activated as a pro-survival signal to restore ER homeostasis. However, persistent stress results in switching of the UPR from a pro-survival signal to a pro-apoptotic signal and then to cell death if the damage becomes irreversible [49]. Our data demonstrate that TMUV infection induced robust and persistent expression of CHOP. Moreover, we found that TMUV infection activated the caspase-3 protease in BHK-21 cells, which is consistent with a previous study reporting that TMUV triggers apoptosis and activates the caspase-3 cascade in duck embryo fibroblasts (DEFs) as well as in Vero and BHK cells [50]. Indeed, activation of caspase and apoptosis appears to be characteristic of flavivirus infection. For example, WNV infection triggers caspase-3 activation and apoptosis both in vitro and in vivo [51]. JEV also initiates the caspase-3 cascade and apoptosis in cultured cells [52], and DENV-induced apoptosis has been reported in diverse types of cells, including brain, epithelial and liver cells [53].

The transcription factor sXBP1 binds to the UPR element (UPRE) and to ERSEs in the promoter regions of target genes. sXBP1 induces a wide range of genes involved in the process of ER-associated degradation (ERAD), by which the ER directs the degradation of misfolded or inappropriate proteins. sXBP1 can also regulate genes encoding chaperones responsible for protein folding [54]. Thus, the IRE1 pathway restores ER homeostasis by enhancing protein-folding capacity and misfolded protein degradation [4]. Previous studies have shown that activation of the IRE1 pathway reduced flavivirus-induced cytotoxicity and allowed the virus to replicate more efficiently [4, 44]. During the UPR, expression of the p58^IPK^ gene is controlled by sXBP1 and connects two of the UPR branches, IRE1 and PERK. In turn, p58^IPK^ interacts with PERK and is serves as an inhibitor of eIF2α [55]. Our data suggest that sXBP1 mRNA begins to accumulate starting at 24 h post-infection, and we also detected induction of p58^IPK^ in TMUV-infected cells at this time point. The functional consequence is that PERK-mediated eIF2α phosphorylation is suppressed and that the IRE1 pathway is triggered by the accumulation of viral proteins and the formation of mature viral particles, consistent with a previous study [3].

According to our data, ATF6 expression and ATF6-mediated regulation of chaperones fluctuated from 12 h to 48 h post-infection. The results reveal that activation of the ATF6 pathway was transient and occurred early in TMUV infection (from 12 h to 24 h post-infection), suggesting that ATF6 pathway activation may not play a major role in TMUV-induced UPR [37]. The observation that the ATF6 pathway was transiently activated was consistent with a previous study of DENV. However, we report here that this activation occurred in the early stage of TMUV infection and ceased prior to activation of the IRE1 pathway. Peña and Harris reported that in DENV-infected cells, ATF6 induction was preceded by activation of the IRE1 pathway [3], and several different viruses, such as WNV and DENV, activate the ATF6 pathway to promote viral replication by regulating signal transduction and innate immune responses [30, 38]. Nevertheless, further investigation is needed to determine whether temporary activation of ATF6 affects TMUV replication during infection.

To explore the UPR in TMUV-infected DF-1 cells, expression of target genes related to the three UPR pathways was assessed, and the results showed that the IRE1 pathway was activated during the middle stage of infection (24 h postinfection) and that the ATF6 pathway was slightly activated in the late stage of infection (48 h post-infection). In contrast, no significant induction of ATF4 was observed in TMUV-infected DF-1 cells at any of the time points evaluated. Thus, the PERK pathway is possibly maintained in an inactive state, enabling translation of viral and cellular genes. However, Vattem and Wek et al. reported that the level of ATF4 expression might be induced by a translational mechanism [56]. Due to the lack of availability of antibodies against chicken UPR factors, the protein levels of upstream phospho-eIF2α and downstream ATF4 were not examined. Thus, such a conclusion cannot be drawn at this time.

## Conclusions

The present study is the first report that TMUV infection is able to activate the PERK, IRE1 and ATF6 signalling pathways of the UPR in a time-dependent manner. The PERK and ATF6 pathways are activated in the early stage of TMUV infection and the IRE1 pathway in the middle stage. Furthermore, the ATF6 pathway is activated transiently and then shut down before activation of the IRE pathway. UPR induction was also observed in TMUV-infected DF-1 cells, primarily through the IRE1 pathway, with some induction via the ATF6 pathway. These findings pave the way for further exploration of the roles that ER stress and UPR play during TMUV infection.

## Methods

### Cells, viruses and antibodies

The baby hamster kidney (BHK-21) and DF-1 cells were obtained from American Type Culture Collection. BHK-21 cells were cultured in RPMI-1640 (HyClone) containing 10% foetal calf serum (FCS, HyClone). DF-1 cells were grown in DMEM (Dulbecco’s Modified Eagle’s medium, Gibco) with 10% FCS (HyClone). TMUV strain JS804 used in this study was isolated from an affected goose with ovary haemorrhage and an enlarged spleen and propagated in BHK-21 cells [1]. Viral titres were determined using a plaque-forming assay, as previously described [57]. Antibodies against phospho-eIF2α, eIF2α, anti-CHOP, p58^IPK^, calreticulin, ATF4 and PDI were obtained from Cell Signaling Technology. The anti-ATF6 antibody was purchased from Santa Cruz Biotechnology. Antibodies against GRP94, GRP78, GADD34, calnexin and a horseradish peroxidase (HRP)-labelled goat anti-mouse IgG antibody were obtained from Abcam. An HRP-labelled goat anti-rabbit IgG antibody was obtained from Invitrogen. The BeyoECL Plus Kit was purchased from Beyotime.

### RNA extraction and real-time RT-PCR detection

Cells were harvested and lysed by Trizol reagent. Total RNA was then extracted by using RNA Purification Kit (Invitrogen) according to the manufacturer’s instructions. 1 μg of total RNA was used to synthesize complementary DNA by using RT SuperMix for qPCR (+gDNA wiper, Vazyme). Target genes were detected by real-time quantitative (q) PCR. Table 1 shows the primers used in this study.

**Table 1:** Primers used in this study

Primer name	Sequence	Primer name	Sequence
GRP78-F	5’-TCATCGGACGCACTTGGAA-3’	Calreticulin-F	5’-GGAGCCTGCCGTCTACTTC-3’
GRP78-R	5’-TAGTGAGAACCATGGCAGAA-3’	Calreticulin-R	5’-GGTCTGGCCCTTGTTACTGA-3’
GRP94-F	5’-CCGAGTTTGATGGGAAGAGGTT-3’	ERp57-F	5’-CTAGGACTGCCGATGGGATTGT-3’
GRP94-R	5’-GGCCACAAGAGCACAAGGAGAT-3’	ERp57-R	5’-AGTTGCTGGCTGCTTTTAGGAA-3’
ATF4-F	5’-AGCAAAACAAGACAGCAGCCACTA-3’	PDI-F	5’-CCCCGGAGGAGGAGGACAAC-3’
ATF4-R	5’-TTGCCTTACGGACCTCCTCTATCA-3’	PDI-R	5’-CACACCACGGGGCATAGAAC-3’
GADD34-F	5’-AGCAGCTGACCGAGGCAAGAG-3’	CAS3-F	5’-GCCCAAACTCTTCATCATTC-3′
GADD34-R	5’-TTAGGGGCGGTCCAAGGTGA-3’	CAS3-R	5’-TCGGCTTCCACTGGTATCTT-3’
CHOP-F	5’-AAGAGGAAGATCAAGGAAGAACTA-3’	CAS8-F	5’-CCTCATCAATCGGCTGGAC-3’
CHOP-R	5’-CCATGCGGTCAATCAGAG-3’	CAS8-R	5’-ATGACCCTGTAGGCAGAAACC-3’ [58]
XBP1-F	5’-GAGAAGGCGCTGCGGAGGAAACTG-3′	CAS9-F	5’-CTCGAGGCAGGGACTTAGACA-3′
XBP1-R	5’-GAGAAAGGGAGGCTGGTAAGGAAC-3′	CAS9-R	5’-AAACTTGACACGGCATCCA-3′ [59]
P58-F	5’-AGATGGCGACCCTGATAACTA-3′	BHK-GAPDHF	5’-ACTTGGCACATGTCTGTATGC-3′
P58-R	5’-GACTGGGCTTCCTTCTCTTC-3′	BHK-GAPDHR	5’-CACCAGCATCACCCCATTT-3’
Calnexin-F	5’-TGCCGAGCCAGGTGTAGTG-3’	DF1-GRP94-F	5’-CTGAGAAGTTTGCCTTTCAAGCAG-3’
Calnexin-R	5’-CCTCTTCATCCCCCTTGTTCTT-3’	DF1-GRP94-R	5’-GCTCCTCATTACCAGCAAGAGCAT-3’ [60]
DF1-uXBP1-F	5’-CAGCACTCAGACTACGTGTTCCTCTG-3′	DF1-sXBP1-F	5’-GCTGAGTCCGCAGCAGG-3′
DF1-uXBP1-R	5’-CTGCCATCAGAATCCATGTG-3’ [60]	DF1-sXBP1-R	5’-CTGCCATCAGAATCCATGTG-3’ [60]
DF1-ATF4-F	5’-CAATTGGCTCGCTGTGGACAGTTT-3′	DF1-GRP78-F	5’-TGTAGCCTATGGTGCAGCTGTTCA-3′
DF1-ATF4-R	5’-ACGGTGGCTTCCAGATGTTCCATA-3’ [60]	DF1-GRP78-R	5’-ATGCCAAGTGTCAGAGGACACACA-3’ [60]
DF1-ACTINF	5’-CTGTGCCCATCTATGAAGGCTA-3′		
DF1-ACTINR	5’-ATTTCTCTCTCGGCTGTGGTG-3′		

### Western blot analysis

Cells infected with TMUV (multiplicity of infection (MOI) = 3) or mock infected were lysed using cell lysis buffer for western blotting and IP containing 1 mM phenylmethylsulfonyl fluoride (PMSF; Beyotime). Protein concentrations were determined by using BCA method (Beyotime). Equal amounts of protein were separated by 12% SDS-PAGE (sodium dodecyl sulfate polyacrylamide gel electrophoresis), followed by transferring proteins from gel to PVDF (polyvinylidene difluoride) membranes using the wet transfer method. The membranes were then blocked with 5% bovine serum albumin (BSA) in PBST (0.5% Tween-20 in phosphate-buffered saline) at 37 °C for 2 h and subsequently incubated overnight with diluted primary antibodies at 4 °C. The membranes were washed by PBST for three times and incubated with secondary antibodies for 1 h at 37 °C. After further washing, the protein bands were detected using a BeyoECL Plus Kit (Beyotime).

### Treatment of BHK-21 cells with a PERK inhibitor

BHK-21 cells were treated with 1 μM of the PERK inhibitor GSK2606414 (Selleck) for 1 h at 37 °C. Then GSK2606414 treated cells were infected with TMUV or mock infected as described above and cultured in fresh medium with 1 μM of GSK2606414 or the corresponding concentration of DMSO (solvent dimethylsulfoxide). Mock-infected cells were used as a control. At 24 h post-infection, cells were harvested and analyzed by western blot.

### Cell viability

BHK-21 cells were treated with GSK2606414 as described above, and the Cell Counting Kit-8 (Dojindo) was used to measure cell viability according to the manufacturer’s protocol.

### Measurement of caspase activity

BHK-21 cells were infected with TMUV or mock infected as described above; mock-infected cells were used as a control. At 12 h, 24 h, 36 h and 48 h post infection, Cells were harvested for measurement of caspase-3, − 8 and − 9 activities using assay kits (Beyotime). Caspase activity is presented as a percentage of the control.

### Dual luciferase reporter assay

Cells were grown in 6-well plates one day before transfection. On the day of transfection, 2 μg of pGM-ATF6-Lu or pGM-ERSE-Lu (pGM-Lu vector, Genomeditech) and 1 μg of pRL-TK vector (Promega) were mixed with Lipofectamine 2000 (Invitrogen) and added to BHK-21 cells. At 48 h post transfection, cells were infected with TMUV (MOI = 3) or mock-infected. After another 12 h, 24 h, 36 h and 48 h, the cells were lysed using a dual luciferase reporter gene assay kit (Beyotime), and activities of luciferase and Renilla luciferase were measured separately using the GloMax-Muti detection system (Promega). The relative fold induction of luciferase activity was calculated.

### Data analysis

Data were analysed with Student’s t test. The software Statistical Package for the Social Sciences (SPSS) was used. The *P* value less than 0.05 was considered significant.

## Abbreviations

ATF4: Activating transcription factor 4
ATF6: Activating transcription factor 6
BHK: Baby hamster kidney cells
BSA: Bovine Serum Albumin
CHOP: C/EBP-homologous protein
CSFV: Classical swine fever virus
DENV: Dengue virus
eIF2α: eukaryotic translation initiation factor 2α
ER: Endoplasmic reticulum
ERAD: Endoplasmic reticulum-associated degradation
ERp57: Endoplasmic reticulum protein 57 kDa
ERSE: Endoplasmic reticulum stress-response element
GADD34: Growth arrest and DNA damage 34
GRP78: Glucose-related protein 78
GRP94: Glucose-related protein 94
HRP: Horseradish Peroxidase
IRE1: Inositol-requiring enzyme 1
JEV: Japanese encephalitis virus
MOI: multiplicity of infection
p58^IPK^: Protein kinase inhibitor p58
PBS: Phosphate buffered saline
PBST: Phosphate buffered saline with tween-20
PDI: Protein disulfide isomerase
PERK: Protein kinase RNA-like endoplasmic reticulum kinase
PMSF: Phenylmethanesulfonyl fluoride
RT-PCR: Reverse transcription polymerase chain reaction
SD: Standard deviation
SDS-PAGE: Sodium dodecyl sulfate polyacrylamide gel electrophoresis
TBEV: Tick-borne encephalitis virus
TMUV: Tembusu virus
UPR: Unfolded protein response
USUV: Usutu virus
WNV: West nile virus
WNV_KUN_: West nile virus Kunjin strain
XBP1: X-boxing binding protein-1
